# Dietary Inflammatory Index and Biomarkers of Lipoprotein Metabolism, Inflammation and Glucose Homeostasis in Adults

**DOI:** 10.3390/nu10081033

**Published:** 2018-08-08

**Authors:** Catherine M. Phillips, Nitin Shivappa, James R. Hébert, Ivan J. Perry

**Affiliations:** 1 HRB Centre for Diet and Health Research, School of Public Health, University College Cork, Western Gateway Building, Western Rd., Cork, Ireland; i.perry@ucc.ie; 2 HRB Centre for Diet and Health Research, School of Public Health, Physiotherapy and Sports Science, University College Dublin, Belfield, Dublin 4, Ireland; 3 Cancer Prevention and Control Program, University of South Carolina, Columbia, SC 29208, USA; shivappa@mailbox.sc.edu (N.S.); jhebert@mailbox.sc.edu (J.R.H.); 4 Department of Epidemiology and Biostatistics, Arnold School of Public Health, University of South Carolina, Columbia, SC 29208, USA; 5 Connecting Health Innovations LLC, Columbia, SC 29201, USA

**Keywords:** inflammation, dietary inflammatory index, metabolic syndrome, lipoproteins, adipocytokines, pro-inflammatory cytokines

## Abstract

Accumulating evidence identifies diet and inflammation as potential mechanisms contributing to cardiometabolic risk. However, inconsistent reports regarding dietary inflammatory potential, biomarkers of cardiometabolic health and metabolic syndrome (MetS) risk exist. Our objective was to examine the relationships between a food frequency questionnaire (FFQ)-derived dietary inflammatory index (DII^®^), biomarkers of lipoprotein metabolism, inflammation and glucose homeostasis and MetS risk in a cross-sectional sample of 1992 adults. Energy-adjusted DII (E-DII) scores derived from an FFQ were calculated. Lipoprotein particle size and subclass concentrations were measured using nuclear magnetic resonance (NMR) spectroscopy. Serum acute-phase reactants, adipocytokines, pro-inflammatory cytokines and white blood cell (WBC) counts were determined. Insulin resistance was calculated by homeostasis model assessment (HOMA-IR). Our data indicate that a more pro-inflammatory diet, reflected by higher E-DII scores, was associated with potentially pro-atherogenic lipoprotein profiles characterised by increased numbers of large very low density lipoprotein (VLDL), small dense low density lipoprotein (LDL) and high density lipoprotein (HDL) particles and less large LDL and HDL particles (all *p* < 0.001). Inflammatory profiling identified a range of adverse phenotypes among those with higher E-DII scores, including higher complement component C3 (C3), C-reactive protein (CRP), (both *p* < 0.05), interleukin 6 (IL-6) and tumour necrosis factor (TNF)-α concentrations, higher WBC counts and neutrophil to lymphocyte ratio (NLR) and lower adiponectin levels (all *p* < 0.001). MetS risk was increased among those with higher E-DII scores (OR 1.37, 95% CI (1.01, 1.88), *p* < 0.05), after adjusting for potential confounders. In conclusion, habitual intake of a more pro-inflammatory diet is associated with unfavourable lipoprotein and inflammatory profiles and increased MetS risk.

## 1. Introduction

The metabolic syndrome (MetS) is a multifactorial condition characterized by a range of metabolic abnormalities including insulin resistance, dyslipidaemia, hypertension and abdominal obesity that is associated with an increased risk of type 2 diabetes mellitus (T2DM), cardiovascular disease (CVD) and atherosclerosis [1,2,3,4]. Recent analysis of the National Health and Nutrition Examination Survey (NHANES) 2007–2014 data revealed that prevalence of MetS among US adults was 34.3%, increasing to almost 55% of adults aged 60 years or over [5]. Thus, MetS represents a major public health concern. Low-grade, systemic inflammation also has been recognised as an important characteristic of the MetS [6,7,8,9,10,11]. Accumulating evidence suggests inflammation as a potential mechanism linking diet and cardiometabolic risk [12]. Diet is an important moderator of inflammation, with certain foods and nutrients capable of eliciting immunomodulatory effects [13,14]. In recent years the Dietary Inflammatory Index (DII^®^) was developed to characterize an individual’s diet on a continuum from maximally anti- to pro-inflammatory [15]. Limited data exist regarding the associations between dietary inflammatory potential, circulating biomarkers of cardiometabolic health and MetS risk. Thus far, the DII has been associated with C-reactive protein (CRP) [16,17,18], interleukin (IL)-6 [19,20], tumour necrosis factor (TNF)-α [19], fibrinogen [21] and more recently with white blood cell (WBC) counts [22]. To date, six studies have examined the association between the DII and MetS risk, with inconsistent relationships identified [16,18,23,24,25,26]. A recent meta-analysis confirmed that individuals with the highest DII scores and thus the most pro-inflammatory diet, showed a 36% increased risk of CVD incidence and mortality, relative to those with the lowest DII scores [27]. However, the relationship between DII and intermediate biomarkers of cardiometabolic health remain largely unknown. Therefore, the main objectives of this paper were to examine whether higher DII scores, reflecting a more pro-inflammatory diet, are associated with unfavourable cardiometabolic health profiles, characterized by biomarkers of inflammation, lipoprotein metabolism and glucose homeostasis and increased MetS risk.

## 2. Materials and Methods

### 2.1. Study Design and Subject Recruitment

The Cork and Kerry Diabetes and Heart Disease Study (Phase II) was a single-centre, cross-sectional study conducted between 2010 and 2011 [28]. A population representative random sample was recruited from a large primary care centre in Mitchelstown, County Cork, Ireland (Mitchelstown cohort, clinical trials.gov identifier NCT03191227). Full details have been published elsewhere [28] but in brief Mitchelstown cohort participants were randomly selected from all registered attending patients in the 50–69 year age group. In total, 3807 potential participants were selected from the practice list. Following exclusion of duplicates, deaths and ineligibles, 3043 were invited to participate in the study and, of these, 2047 (49.2% male) completed the questionnaire and physical examination components of the baseline assessment (response rate 67%). Ethics committee approval conforming to the Declaration of Helsinki was obtained from the Clinical Research Ethics Committee of University College Cork. All participants provided written informed consent. Following exclusion of individuals without food frequency questionnaire (FFQ) data the remaining 1992 participants were used in the analyses. A flow chart outlining the subject selection for the current analysis of the Mitchelstown cohort is presented in Supplemental Figure S1.

### 2.2. Clinical and Anthropometric Data

All participants attended the clinic in the morning after an overnight fast (minimum 8 h). Fasting blood samples were taken on arrival. Participants then completed a General Health Questionnaire (GHQ), the International Physical Activity Questionnaire (IPAQ) [29] and the FFQ [30,31]. Data on age, gender and medication use was gathered through the self-completed GHQ. The presence of cardiovascular disease (CVD) was obtained from the GHQ by asking study participants if they had been diagnosed with any one of the following seven conditions: Heart Attack (including coronary thrombosis or myocardial infarction), Heart Failure, Angina, Aortic Aneurysm, Hardening of the Arteries, Stroke, or any other Heart Trouble. Subjects who indicated a diagnosis of any one of these conditions were classified as having CVD. T2DM was defined according to the American Heart Association guidelines of fasting plasma glucose (FPG) ≥ 7 mmol/L or doctor diagnosed diabetes. Blood pressure was measured according to the European Society of Hypertension Guidelines using an OMRON M7 Digital BP monitor (OMRON Healthcare Europe B.V, Hoofddorp, The Netherlands) on the right arm, after a 5-min rest in the seated position. The average of the second and third measurements was used for analyses. MetS was defined according to the National Cholesterol Education (NCEP) Adult Treatment Panel III (ATP III) [32]. Anthropometric measurements were recorded with calibrated instruments according to a standardised protocol. Body weight was measured in kilograms without shoes, to the nearest 100 g, using a Tanita WB100MA weighing scales (Tanita Corporation, IL, USA). Height was measured in centimetres to 1 decimal place using a Seca Leicester height gauge (Seca, Birmingham, UK). Weight and height measurements were used to calculate body mass index (BMI). Waist circumference (defined as mid-way between lowest rib and iliac crest) was measured in centimetres to 1 decimal place using a Seca 200 measuring tape (Seca, Birmingham, UK). The average of two measures was used for analyses. 

### 2.3. Diet and Lifestyle Data

Physical activity levels were assessed using the short form IPAQ [29] and were defined as low, moderate and high. Smoking status was defined as never, former and current smokers. Alcohol consumption included questions based on weekly intake to define never, moderate and heavy drinkers. Diet was assessed using a modified version of the self-completed European Prospective Investigation into Cancer and Nutrition EPIC FFQ (UK arm) [31]. Adapted by the Irish National Nutrition Surveillance Centre to reflect the Irish diet, this FFQ has been validated for use in the Irish population [30]. Information on the frequency of consumption of food items during the past 12 months was collected. The daily intake of energy and nutrients was computed from FFQ data using a tailored computer program (FFQ Software Ver 1.0; developed by the National Nutrition Surveillance Centre, School of Public Health, Physiotherapy and Sports Science, University College Dublin, Belfield, Dublin 4, Ireland), which linked frequency selections with the food equivalents in McCance and Widdowson Food Tables [33].

### 2.4. Dietary Inflammatory Index

To compute the Energy-adjusted DII (E-DII) score, dietary information for each study participant is first linked to a regionally representative database that provides a global estimate of mean intake for each of the 45 parameters, (i.e., foods, nutrients and other food components) along with its standard deviation considered in the DII definition [15]. These parameters then are used to derive the participant’s exposure relative to the standard global mean as a z-score, derived by subtracting the mean of the energy-adjusted regionally representative database from the amount reported and dividing this value by the parameter’s standard deviation. These z-scores are converted to proportions (i.e., with values ranging from 0 to 1) and then centring by doubling and subtracting 1. Clinical interpretation remains clear with these additional steps and inappropriate weighting is avoided and higher (i.e., more positive) DII scores invariably represent more pro-inflammatory diets. The resulting value is then multiplied by the corresponding food parameter effect score (derived from a literature review on the basis of 1943 peer-reviewed articles [15]. All of these food parameter-specific E-DII scores are then summed to create the overall DII score for every subject in the study. A total of 26 of the 45 possible food parameters were used for DII calculation based on the FFQ in this study and these were as follows: carbohydrate, protein, fat, alcohol, fibre, cholesterol, saturated fat, mono-unsaturated fat, poly-unsaturated fat, niacin, thiamin, riboflavin, vitamin-B12, vitamin-B6, iron, magnesium, zinc, selenium, vitamin A, vitamin C, vitamin D, vitamin E, folic acid, onion, garlic and tea.

### 2.5. Biological Analyses

Plasma and serum were prepared from fasting blood samples from each subject. Fasting plasma glucose (FPG), serum total, HDL cholesterol (HDL-C), LDL cholesterol (LDL-C) and triglyceride (TAG) concentrations were measured by Cork University Hospital Biochemistry Laboratory using fresh blood samples. FPG concentrations were determined using a glucose hexokinase assay and serum lipids were analysed using enzymatic colorimetric tests (Olympus Life and Material Science Europa Ltd., Lismeehan, Co., Clare, Ireland) on an Olympus 5400 automatic analyser (Olympus Diagnostica Gmbh, Hamburg, Germany). Serum insulin, C-reactive protein (CRP), tumour necrosis factor (TNF)-α concentrations, interleukin 6 (IL-6), adiponectin (ACDC), leptin and resistin were determined using a biochip array system (Evidence Investigator; Randox Laboratories, Antrim, UK). C3 was determined by immunoturbidimetric assay (Rx Daytona; Randox Laboratories, Antrim, UK). WBC counts were determined by flow cytometry technology as part of a full blood count by the Cork University Hospital Haematology Laboratory using fresh blood samples. Homeostasis model assessment HOMA-IR, a measure of insulin resistance, was calculated as ((fasting plasma glucose x fasting serum insulin)/22.5). Quantitative insulin-sensitivity check index (QUICKI), a measure of insulin sensitivity, was calculated as (1/(log insulin0 + log glucose0)).

### 2.6. Lipoprotein Particle Profiling

Lipoprotein subclass particle concentrations and average VLDL, LDL and HDL particle diameters were measured on serum specimens by NMR spectroscopy at LipoScience Inc. (Raleigh, NC, USA). LDL, HDL and VLDL subclasses were quantified based on the amplitudes of their spectroscopically distinct lipid methyl group NMR signals [34]. Weighted-average VLDL, LDL and HDL particle sizes (in nanometre diameter units) were computed as the sum of the diameter of each subclass multiplied by its relative mass percentage as estimated from the amplitude of its NMR signal. The following 9 subclass categories were investigated: large VLDL (including chylomicrons, if present) (>60 nm), medium VLDL (42 to 60 nm), small VLDL (29 to 42 nm), large LDL (20.5 to 23 nm), small LDL (18 to 20.5 nm), large HDL (9.4 to 14 nm), medium HDL (8.2 to 9.4 nm) and small HDL (7.3 to 8.2 nm). Particle concentrations are expressed as nanomoles per litre (VLDL and LDL) and micromoles per litre (HDL). A Lipoprotein Insulin Resistance score (LP-IR), ranging from 0 (least) to 100 (most) insulin resistant, which is a weighted combination of the 6 lipoprotein subclass and size parameters most closely associated with IR, was calculated [35].

### 2.7. Statistical Analysis

Statistical analysis was conducted using IBM Statistics version 20.0 for Windows (SPSS Inc., Chicago, IL, USA). Continuous variables are expressed as means ± SEM and categorical variables as percentages. Inflammatory markers and lipoprotein parameters were assessed for normality of distribution and skewed variables were normalised by log_10_ transformation as appropriate. Differences between groups were analysed by independent *t*-tests or Mann Whitney U tests for continuous variables and by Chi-Square test for categorical variables. A composite inflammatory score was calculated based on tertiles of C3, CRP, IL-6, TNF-α, leptin, adiponectin and WBC counts. Tertiles 1–3 for each marker (where 1 indicates the least pro-inflammatory level and 3 the most pro-inflammatory level, reverse scored for adiponectin) were summed. Scores ranged from 1 to 15. Logistic regression was used to determine associations between the E-DII, inflammatory and lipoprotein profiles, composite inflammatory score (stratified by median level) and MetS status. Multivariate logistic regression analysis was performed including age, gender, BMI, waist circumference, physical activity, smoking status, alcohol consumption and use of anti-inflammatory and lipid lowering medication as confounding factors. For all analyses, a *p*-value of <0.05 was considered significant.

## 3. Results

### 3.1. Clinical and Demographic Characteristics Stratified by E-DII Median

A total of 1992 participants with complete data were included in the current investigation. Median (SD) and range of the E-DII in the Mitchelstown cohort were −1.40 (1.50) and −5.10 to +3.68. Clinical and demographic characteristics of the cohort according to E-DII median are presented in Table 1. Individuals with higher (more positive) E-DII scores and thus a more pro-inflammatory diet, were more likely to be slightly younger males with larger waist circumference, lower HDL-C concentrations and higher LDL-C, triglyceride and glucose concentrations and systolic blood pressure (SBP) (all *p* < 0.05), relative to those with a less inflammatory diet (<median E-DII scores). Regarding lifestyle behaviours, those with a more pro-inflammatory diet were more likely to be current smokers and more sedentary. Prevalence of MetS was greater among those with higher E-DII scores (>median E-DII scores), whereas no differences in prevalence of T2DM, CVD or hypertension or medication use were observed between groups. Logistic regression analysis revealed that MetS risk was 37% higher among those with higher E-DII scores compared to those among the bottom E-DII median (OR 1.37, 95% CI (1.01, 1.88), *p <* 0.05), after adjusting for potential confounders including age, gender, BMI, physical activity, smoking status, alcohol consumption and use of anti-inflammatory and lipid-lowering medication.

### 3.2. Dietary Composition and Food Pyramid Servings

Daily energy intake and dietary macronutrient composition (Table 2) were similar between groups stratified by E-DII median. Examination of daily number of servings based on food pyramid recommendations revealed that those with a more pro-inflammatory diet consumed more servings from each of the shelves of the food pyramid. In particular, the number of servings of fats and high fat/sugar foods was 70% greater compared to those with a more anti-inflammatory diet, whereas the number of servings of fruit and vegetables was 64% lower than their counterparts with lower DII scores. No differences in micronutrient intake (data not shown) were observed between groups. It is important to note that upon further examination intake of refined grains/cereals, red and processed meats and full-fat dairy were higher and consumption of fish, wholegrains and low-fat dairy products were lower among those with a more pro-inflammatory diet (all *p* < 0.001).

### 3.3. Lipoprotein Profiles Stratified by E-DII Median

Lipoprotein particle concentrations and size profiles of the study population according to E-DII median levels are presented in Table 3. A more pro-inflammatory diet was associated with a less favourable lipoprotein profile characterized by larger VLDL, IDL, small dense LDL and HDL particles (all *p* < 0.001) and less large LDL and HDL particles (both *p* < 0.001) compared to those with below-median E-DII scores. These changes translated into larger average VLDL particle size (*p* = 0.003) and smaller average LDL and HDL particle size (both *p* < 0.001). Furthermore, lipoprotein-associated insulin resistance was greater among those with a more pro-inflammatory diet (*p* < 0.001). Logistic regression analysis confirmed increased likelihood of large (>median diameter) VLDL particles (OR 1.28, 95% CI 1.07–1.54, *p* = 0.008), small HDL particle size (OR 1.45, 95% CI 1.21–1.74, *p* < 0.001), small LDL particle size (OR 1.54, 95% CI 1.28–1.84, *p* < 0.001) and high LP-IR scores (>median) (OR 1.24, 95% CI 1.10–1.50, *p* = 0.03), among those with a more pro-inflammatory diet after controlling for age, gender, BMI and medication use. However, these associations were attenuated in the fully adjusted models.

### 3.4. Inflammatory Profiles Stratified by E-DII Median

Concentrations of pro-inflammatory cytokines, acute-phase response proteins, adipocytokines and the WBC profiles of the study population stratified by E-DII median are presented in Table 4. Those with higher E-DII scores displayed a more unfavourable inflammatory profile characterized by higher C3, CRP, (both *p* < 0.05), IL-6, TNF-α concentrations (both *p* < 0.001) and lower adiponectin levels (*p* < 0.001), compared to their counterparts with lower E-DII scores. Greater WBC counts (accounted for by increased neutrophils, monocytes (both *p* < 0.001) and basophils (*p* < 0.05)) and higher NLR (*p* < 0.01) were observed among those with the most pro-inflammatory E-DII scores. Logistic regression analysis confirmed that increased risk of adverse (>median) concentrations of CRP (OR 1.35, 95% CI 1.05–1.72, *p* = 0.018), IL-6 (OR 1.47, 95% CI 1.14–1.89, *p* = 0.003), TNF-α OR 1.55, 95% CI 1.22–1.99, *p* < 0.001) and WBC counts (OR 1.40, 95% CI 1.10–1.79, *p* = 0.009) persisted among those with the most pro-inflammatory diet even after controlling for potential confounders. Consequently, the composite inflammatory score was also greater among those with a more inflammatory diet and the risk of having a high composite inflammatory score was 40% higher (OR 1.40, 95% CI 1.02–1.94, *p* = 0.04 in the fully adjusted model) compared to those with a more anti-inflammatory diet.

## 4. Discussion

In this study, we demonstrated that individuals with higher E-DII scores displayed several features of the MetS including lower HDL-C concentrations, higher triglyceride and fasting blood glucose concentrations, elevated SBP and larger waist circumference, relative to those with a less inflammatory diet (<median E-DII scores). These unfavourable profiles translated into an increased risk of MetS among those with higher E-DII scores. Furthermore, lipoprotein and inflammatory marker profiling identified a range of adverse metabolic features including greater numbers of large VLDL particles and small dense LDL and HDL particles in combination with elevated concentrations of C3, CRP, IL-6, TNF-α and WBC counts, higher NLR and lower adiponectin levels, among those with higher E-DII scores compared to their counterparts with lower E-DII scores.

Associations between dietary inflammatory potential and MetS components including elevated blood pressure [25,26], raised triglyceride and low HDL-C concentrations [25], glucose intolerance [18], waist to hip ratio and waist circumference [24,26] have been reported. However inconsistent reports regarding the association between dietary inflammatory potential and MetS risk exist [18,23,24,25,26]. Most investigations have failed to identify a significant relationship; however, differences in MetS definitions and prevalence, study size, dietary assessment tools and number of food items available for DII calculation as well as inherent population differences in the inflammatory potential of habitual diet, may be partly accountable. In the Seguimiento University of Navarro (SUN) cohort of 6851 subjects with 8.3 years follow-up, no association was found between DII and incident MetS [25]. Mazidi et al., in a recent examination of data from the NHANES (2005–2012) reported increased levels of all MetS components (decreased for HDL-C) across increasing DII quartiles, resulting in 65% greater risk of MetS among those in the fourth quartile relative to the first (and least pro-inflammatory) quartile [17]. We report a similar MetS risk (OR 1.37 comparing top 50th percentile of DII score to bottom 50th percentile) to that reported in the French Supplementation en Vitamines et Mineraux Antioxydants (SU.VI.MAX) study (OR 1.39 comparing quartile 4 of DII score to quartile 1) [24]. It is worth noting that the studies which reported increased MetS risk with higher DII scores also report similar MetS prevalence and participant age range. Furthermore, considering that the median DII score was lower (less pro-inflammatory) in the Irish cohort compared to the French and American cohorts (−1.28 vs. 0.58 and 0.44, respectively), may indicate that the increased MetS risk observed in the current study may underestimate the MetS risk conferred by more pro-inflammatory diets habitually consumed in other populations. It is also noteworthy that individuals with higher E-DII scores had larger waist circumference, were more sedentary and, despite no differences in total energy intake, they appeared to consume more servings from all food pyramid shelves, with the exception of fruit and vegetables, relative to those with <median E-DII scores. Further examination revealed higher intake of refined grains/cereals, red and processed meats and full-fat dairy and lower consumption of fish, wholegrains and low-fat dairy products among those with a more pro-inflammatory diet. Although we included anthropometric and lifestyle factors in our models the role of other unmeasured diet or lifestyle behaviours cannot be excluded.

Chronic, low-grade, systemic inflammation is a pathological feature of several chronic conditions including MetS, T2DM and CVD [12]. Increasing evidence suggests that some foods, food components and nutrients may modulate inflammatory status [13,14]. Supporting that concept are the findings that the DII has been associated with circulating concentrations of CRP [16,17,18], IL-6 [19,20], TNF-α [19] and WBC counts [22]. Examination of pro-inflammatory cytokines, acute-phase response proteins, adipocytokines and WBC profiles of the study population stratified by E-DII median in the present work revealed higher C3, CRP, IL-6 and TNF-α concentrations and WBC counts (due to elevated neutrophils, monocytes and basophils), higher NLR and lower adiponectin levels among those with higher E-DII scores (top 50th percentile) relative to their counterparts with lower E-DII scores. The magnitude of the difference observed in CRP levels (11.9%) comparing individuals by median DII scores was greater than that reported between non-cases and cases of type 2 diabetes in the Caerphilly study [36] and CVD events in the Health ABC study [37]. Observed differences in total WBC counts and IL-6 levels (11%) were greater than those reported between non-cases and cases of CVD and non-cases and cases of type 2 diabetes in the Caerphilly study, respectively [36]. Similarly, the observed difference in adiponectin concentrations (10.6%) between those with the most anti-inflammatory and most pro-inflammatory diets was similar to that reported between CVD cases and non-cases in the Cardiovascular Health Study [38] and exceeded that reported between patients with and without CVD events in the British Regional Heart Study [39]. Collectively, these findings suggest potentially clinically significant differences in inflammatory profiles and furthermore that the observed association between DII and MetS may be, at least in part, mediated by sub-clinical inflammation.

A protective effect of an anti-inflammatory diet on CVD incidence over a 10-year follow up of the ATTICA study has been reported [40]. Moreover, a recent meta-analysis confirmed that individuals with the highest DII scores and thus the most pro-inflammatory diet, showed a 36% increased risk of CVD incidence and mortality, relative to those with the lowest DII scores [27]. Tyrovalas et al., examined the association between DII and CVD risk factors (obesity, diabetes, hypertension and hypercholesterolemia) in US adults using data from the NHANES (2007–2012) [41]. They reported a dose-dependent relationship whereby risk of at least one risk factor was 1.5 times higher comparing those with the highest quartile of DII to the lowest quartile of DII. Both triglyceride and LDL-C concentrations are strong predictors of CVD morbidity and mortality. Results from the SU.VI.MAX study provide evidence that higher DII scores are prospectively associated with higher triglyceride and lower HDL-C concentrations [25]. However, no study to date has examined DII in the context of lipoprotein particle subclass. Our findings suggest that a more pro-inflammatory diet was associated with an unfavourable lipoprotein profile characterized by higher numbers of large VLDL particles and small dense LDL and HDL particles and reduced numbers of large LDL and HDL particles relative to those with a more anti-inflammatory diet. These changes translated into larger average VLDL particle size and smaller average LDL and HDL particle size. Small, dense LDL and HDL particles are associated with increased risk for atherosclerosis and premature CVD [42,43,44]. Large VLDL particles are important in terms of CVD risk as they are associated with the pro-atherogenic small, dense LDL phenotype [43]. Relative to LDL particles these large lipid-enriched VLDL particles are more efficiently hydrolysed by lipoprotein lipase, have greater capacity to penetrate the endothelial wall and be preferentially retained in the arterial intima. VLDL particles also may be directly taken up by macrophages (without any modifications like LDL) to create foam cells, the hallmark cells of atherosclerotic plaque. Furthermore, the NLR, a marker of systemic inflammation and strong predictor for CVD risk and mortality [45] was higher among those with higher DII scores. Thus, dietary strategies, such as adopting a more anti-inflammatory diet, may improve dyslipidaemia and attenuate atherogenesis and related cardiometabolic disease. Recent data from a small dietary intervention study (*n* = 65) revealed that a reduction in DII score following a healthy diet (Mediterranean or low-fat diet intervention for 6 months) was associated with reduced IL-6 and triglyceride concentrations [46].

Our study has several strengths including a large number of participants aged 50 to 69 years old with evaluable data and equal representation by gender (49% male); determination of a wide range of biomarkers of lipoprotein metabolism, inflammation and glucose homeostasis; assessment of a wide range of clinical health parameters, diet and lifestyle factors; and a wide range of confounding factors, including medication use. Notwithstanding these strengths several limitations can also be identified. The cross-sectional study design prohibits us from drawing conclusions regarding both causality and the temporal direction of the observed associations between dietary inflammation, unfavourable lipoprotein and inflammatory profiles and MetS. However, it is conceivable that a more inflammatory diet may lead to metabolic perturbations (such as raised inflammatory status and unfavourable lipoprotein profiles), which predispose to adverse metabolic health and conditions such as MetS. Previously, we have demonstrated increased likelihood of metabolic health, regardless of BMI status, among those with favourable lipoprotein and inflammatory profiles [47,48]. Although both genders were equally represented in the cohort, this was not the case when stratified by E-DII. Gender may influence both exposures (food choices) and outcomes (such as waist circumference), thus we included gender as a covariate in our models. Although we controlled for confounding factors, we cannot exclude the possibility that unmeasured confounders may also influence our observations. Moreover, residual confounding arising from imprecise measurement of physical activity and dietary intake should also be considered. The short-form IPAQ may over-estimate physical activity relative to objective measures [49]. Similarly, dietary assessment using an FFQ can introduce dietary reporting biases, such as social desirability and approval [50]. Another potential limitation is the non-availability of information on the remaining 18 food parameters for DII calculation. However, on average, we have had data on 27 food parameters for DII generation and where we have been able to compare across methods no change in the predictive ability of DII when going from 45 to less than 30 food parameters [17,19]. Finally, the generalizability of our findings may be limited. The Mitchelstown cohort comprises adults recruited from a large primary care centre in Mitchelstown, County Cork, Ireland, which includes 8 general practitioners (GP) serving an urban and rural catchment area of approximately 20,000. Around 98% of Irish adults are registered with a GP making it possible, even in the absence of a universal patient registration system, to undertake population-based epidemiological studies that are representative of the general population [51].

## 5. Conclusions

In conclusion, these novel results provide further evidence regarding the relationship between dietary inflammatory potential, intermediate biomarkers of cardiometabolic health and MetS risk. Importantly, they highlight the potential of a more anti-inflammatory diet extends beyond anti-inflammatory effects and may be useful in achieving and maintaining a more favourable cardiometabolic risk profile and reducing likelihood of MetS and CVD. Investigation in other populations is warranted. Improving our understanding of the relationship between the inflammatory capacity of habitual diet and biomarkers of cardiometabolic health is important in the development of more effective dietary interventions and public health policy to reduce inflammation and promote good metabolic health at the population level.

## Figures and Tables

**Table 1:** Characteristics of the Mitchelstown cohort (*n* = 1992) by E-DII median.

Min and Max of E-DII Scores	<Median E-DII −5.10 to −1.28	>Median E-DII −1.28 to 3.68	*p*
E-DII	−2.51 ± 0.02	−0.06±0.03	<0.001
Age (years)	59.9 ± 0.17	59.5±0.17	0.04
Gender (% male)	38.6	59.4	<0.001
BMI (kg/m^2^)	28.40 ± 0.15	28.72 ± 0.15	0.12
Waist (cm)	95.48 ± 0.41	98.32 ± 0.41	<0.001
Total cholesterol (mmol/L)	5.25 ± 0.03	5.31 ± 0.03	0.24
LDL-C (mmol/L)	3.13 ± 0.03	3.22 ± 0.03	0.04
HDL-C (mmol/L)	1.48 ± 0.01	1.42 ± 0.01	<0.001
Triglycerides (mmol/L)	1.34 ± 0.02	1.45 ± 0.03	0.004
FPG (mmol/L)	5.17 ± 0.04	5.21 ± 0.04	0.03
Insulin (µIU/mL)	11.02 ± 0.29	12.10 ± 0.35	0.06
HOMA	2.71 ± 0.09	2.98 ± 0.10	0.06
QUICKI	0.28 ± 0.002	0.27 ± 0.002	0.26
SBP (mm Hg)	128.5 ± 0.52	130.6 ± 0.54	0.005
DBP (mm Hg)	79.9 ± 0.31	80.5 ± 0.31	0.17
Current smokers (%)	12.6	16.7	0.008
Moderate and heavy alcohol consumers (%)	78.9	80.5	0.49
Low intensity physical activity (%)	43.5	52.9	<0.001
Type 2 diabetes (%)	8.9	8.5	0.71
CVD (%)	10.7	10.1	0.66
MetS (%)	21.5	25.4	0.04
Obesity (%)	36.3	34.0	0.14
Hypertension (%)	46.5	43.7	0.24
Anti-inflammatory medication (%)	4.1	4.3	0.82
Lipid lowering medication (%)	6.9	6.4	0.65

Continuous variables are expressed as means ± SEM; categorical variables are expressed as percentages. *p* was derived from Students *t*-tests and non-parametric tests for continuous variables and Chi-Square test for categorical variables. %: percentage; E-DII: Energy-adjusted dietary inflammatory index; DBP: Diastolic blood pressure; LDL-C: LDL cholesterol; HDL-C: HDL cholesterol; FPG: fasting plasma glucose; HOMA: Homeostasis model assessment; QUICKI: Quantitative insulin-sensitivity check index; CVD: cardiovascular disease; MetS: metabolic syndrome.

**Table 2:** Nutritional intake of the study population (*n* = 1992) stratified by E-DII median.

Min and Max of E-DII Scores	<Median E-DII −5.10 to −1.28	>Median E-DII −1.28 to 3.68	*p*
Dietary composition			
Kilocalories	2056 ± 26	2000 ± 25	0.80
Fat (% EI)	33.74 ± 0.22	33.77 ± 0.28	0.06
SFA (% EI)	34.49 ± 0.20	34.57 ± 0.20	0.79
PUFA (% EI)	19.98 ± 0.18	19.90 ± 0.18	0.830
MUFA (% EI)	31.60 ± 0.10	31.53 ± 0.10	0.28
Carbohydrate (% EI)	49.23 ± 0.27	48.64 ± 0.28	0.07
Protein (% EI)	18.42 ± 0.13	18.76 ± 0.14	0.08
Sugar (% EI)	20.57 ± 0.23	20.71 ± 0.25	0.37
Alcohol (% EI)	1.51 ± 0.09	1.65 ± 0.10	0.13
Fibre (% EI)	2.62 ± 0.02	2.57 ± 0.02	0.08
Daily food pyramid shelf servings			
Bread, cereal, potatoes, grain and rice	5.03 ± 0.09	5.51 ± 0.10	<0.001
Fruit and vegetables	9.00 ± 0.18	5.75 ± 0.11	<0.001
Dairy	1.77 ± 0.04	2.11 ± 0.05	<0.001
Meat, fish, poultry and eggs	2.25 ± 0.03	2.55 ± 0.05	<0.001
Fats, high fat/sugar foods and drinks	5.85 ± 0.10	9.95 ± 0.18	<0.001

Values are presented as means ± SEM. *p* values were derived from Students *t*-tests and non-parametric tests. EI; energy intake, MUFA: monounsaturated fatty acids; PUFA: polyunsaturated fatty acids; SFA: saturated fatty acids.

**Table 3:** Lipoprotein profiles of the Mitchelstown cohort (*n* = 1992) according to E-DII median.

Min and Max of E-DII Scores	<Median E-DII −5.10 to −1.28	>Median E-DII −1.28 to 3.68	*p*
Lipoprotein Particle Concentration			
Total TRL (nmol/L)	65.84 ± 1.40	67.60 ± 1.36	0.37
Large VLDL (nmol/L)	2.31 ± 0.136	3.05 ± 0.16	<0.001
Medium VLDL (nmol/L)	26.91 ± 0.79	28.54 ± 0.79	0.14
Small VLDL (nmol/L)	36.62 ± 0.89	36.01 ± 0.83	0.61
IDL (nmol/L)	105.42 ± 2.68	120.66 ± 2.98	<0.001
Total LDL (nmol/L)	1232.54 ± 12.96	1294.55 ± 13.24	0.001
Large LDL (nmol/L)	623.12 ± 10.02	570.03 ± 9.24	<0.001
Small LDL (nmol/L)	504.00 ± 12.92	603.84 ± 13.65	<0.001
Total HDL (mol/L)	38.51 ± 0.20	38.20 ± 0.20	0.27
Large HDL (mol/L)	7.46 ± 0.14	6.53 ± 0.13	<0.001
Medium HDL (mol/L)	13.82 ± 0.20	13.24 ± 0.19	0.04
Small HDL (mol/L)	17.22 ± 0.18	18.42 ± 0.19	<0.001
Lipoprotein Particle Size			
VLDL (nm)	44.61 ± 0.20	45.48 ± 0.22	0.003
LDL (nm)	20.93 ± 0.02	20.81 ± 0.02	<0.001
HDL (nm)	9.35 ± 0.02	9.23 ± 0.02	<0.001
LP-IR score	30.74 ± 0.69	37.08 ± 0.72	<0.001

Values are expressed as means ± SEM. *p* values were derived from Students t-tests and non-parametric tests. IDL: intermediate density lipoprotein; LP-IR: lipoprotein associated insulin resistance; TRL: triglyceride rich lipoproteins.

**Table 4:** Inflammatory profiles of the study population (*n* = 1992) according to dietary inflammation status.

Min and Max of E-DII Scores	<Median E-DII −5.10 to −1.28	>Median E-DII −1.28 to 3.68	*p*
Inflammatory score	7.74 ± 0.12	8.29 ± 0.10	<0.001
C3 (mg/dL)	134.31 ± 0.78	136.90 ± 0.76	0.04
CRP (mg/L)	2.19 ± 0.12	2.45 ± 0.11	0.03
IL-6 (pg/mL)	2.72 ± 0.14	3.02 ± 0.15	<0.001
TNF-α (pg/mL)	6.23 ± 0.08	6.51 ± 0.09	0.001
Adiponectin (ng/mL)	6.05 ± 0.13	5.41 ± 0.13	<0.001
Leptin (ng/mL)	2.85 ± 0.12	2.78 ± 0.10	0.11
Resistin (ng/mL)	5.64 ± 0.10	5.78 ± 0.11	0.50
WBC (109/L)	5.85 ± 0.07	6.14 ± 0.06	0.001
Neutrophils (109/L)	3.23 ± 0.04	3.48 ± 0.04	<0.001
Lymphocytes (109/L)	1.83 ± 0.02	1.86 ± 0.03	0.37
Monocytes (109/L)	0.51 ± 0.005	0.54 ± 0.01	<0.001
Eosinophils (109/L)	0.20 ± 0.004	0.21 ± 0.005	0.06
Basophils (109/L)	0.031 ± 0.001	0.033 ± 0.001	0.03
Neutrophil to lymphocyte ratio	1.89 ± 0.03	2.04 ± 0.03	<0.001

Values are expressed as means ± SEM. *p* values were derived from Students *t*-tests and non-parametric tests. A composite inflammatory score was calculated based on tertiles of C3, C-reactive protein (CRP), interleukin 6 (IL-6), tumour necrosis factor (TNF)-α, leptin, adiponectin and white blood cell (WBC) counts. Tertiles 1–3 for each marker (where 1 indicates the least pro-inflammatory level and 3 the most pro-inflammatory level, reverse scored for adiponectin) were summed. Scores ranged from 1 to 15.

## Supplementary Materials

The following are available online at http://www.mdpi.com/2072-6643/10/8/1033/s1, Figure S1: Flow chart outlining the subject selection for the current analysis of the Mitchelstown cohort.
