# Supplementary material for: Dietary Inflammatory Index and Biomarkers of Lipoprotein Metabolism, Inflammation and Glucose Homeostasis in Adults
Source: Nutrients. 2018 Aug 8;10(8):1033. doi: 10.3390/nu10081033 (PMC6115860; doi:10.3390/nu10081033)

Supplementary Figure 1. Flow chart outlining the subject selection for the current analysis of the Mitchelstown cohort

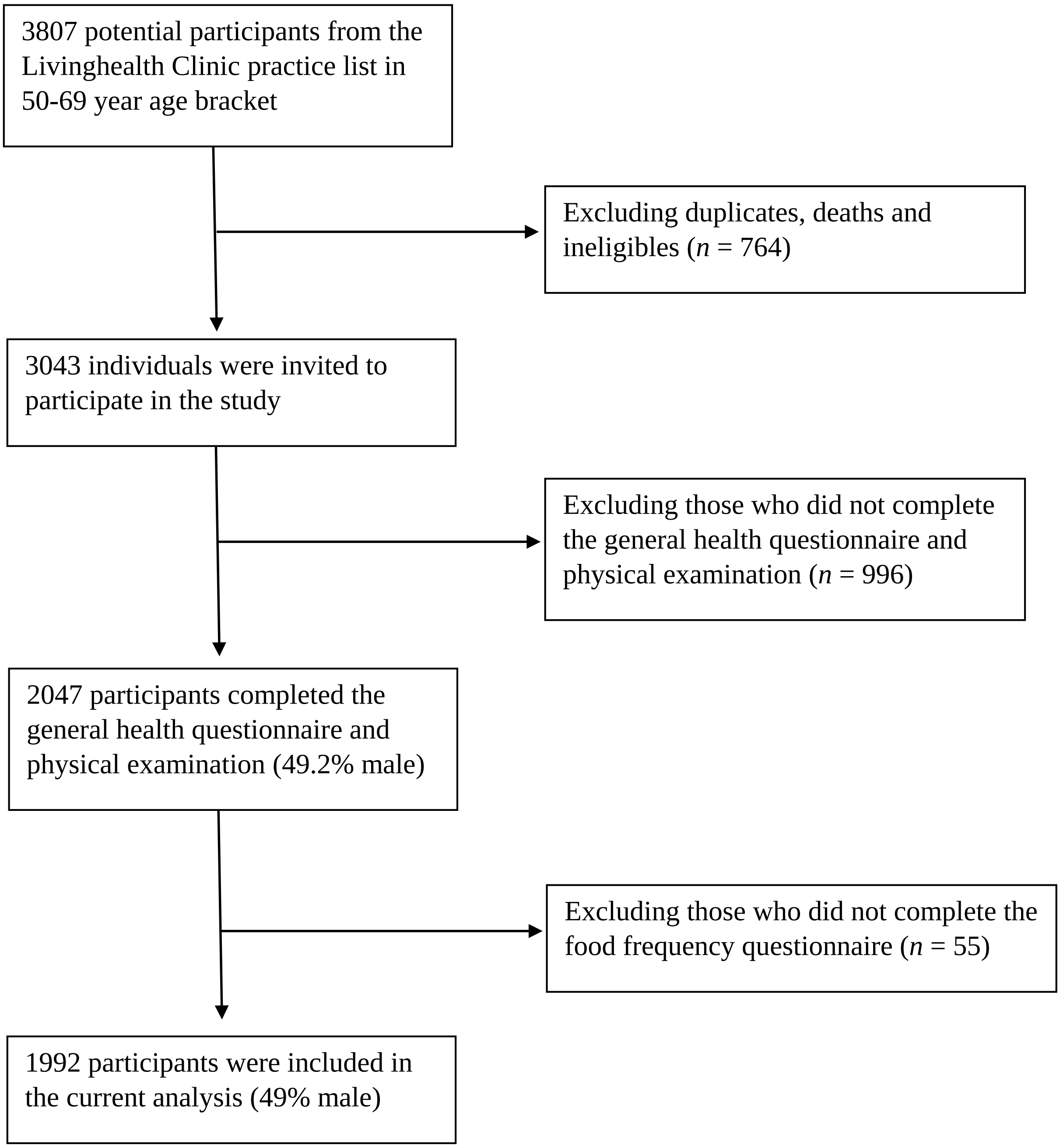

Supplement: Supplementary file 1 [file nutrients-10-01033-s001.pdf]
